# Multiplexed Fc array for evaluation of antigen-specific antibody effector profiles

**DOI:** 10.1016/j.jim.2017.01.010

**Published:** 2017-04

**Authors:** Eric P. Brown, Karen G. Dowell, Austin W. Boesch, Erica Normandin, Alison E. Mahan, Thach Chu, Dan H. Barouch, Chris Bailey-Kellogg, Galit Alter, Margaret E. Ackerman

**Affiliations:** a Thayer School of Engineering, Dartmouth College, Hanover, NH 03755, USA; b Department of Computer Science, Dartmouth College, Hanover, NH 03755, USA; c Ragon Institute of Massachusetts General Hospital, Massachusetts Institute of Technology and Harvard University, Cambridge, MA, USA; d Beth Israel Deaconess Medical Center, Boston, MA, USA

**Keywords:** Antibody, Effector function, ADCC, Phagocytosis, Vaccines, IgG

## Abstract

Antibodies are widely considered to be a frequent primary and often mechanistic correlate of protection of approved vaccines; thus evaluating the antibody response is of critical importance in attempting to understand and predict the efficacy of novel vaccine candidates. Historically, antibody responses have been analyzed by determining the titer of the humoral response using measurements such as an ELISA, neutralization, or agglutination assays. In the simplest case, sufficiently high titers of antibody against vaccine antigen(s) are sufficient to predict protection. However, antibody titer provides only a partial measure of antibody function, which is dependent on both the variable region (Fv) to bind the antigen target, and the constant region (Fc) to elicit an effector response from the innate arm of the immune system. In the case of some diseases, such as HIV, for which an effective vaccine has proven elusive, antibody effector function has been shown to be an important driver of monoclonal antibody therapy outcomes, of viral control in infected patients, and of vaccine-mediated protection in preclinical and clinical studies. We sought to establish a platform for the evaluation of the Fc domain characteristics of antigen-specific antibodies present in polyclonal samples in order to better develop insights into Fc receptor-mediated antibody effector activity, more fully understand how antibody responses may differ in association with disease progression and between subject groups, and differentiate protective from non-protective responses. To this end we have developed a high throughput biophysical platform capable of simultaneously evaluating many dimensions of the antibody effector response.

## Highlights

• High-throughput array-based characterization platform for polyclonal antibodies.
• Development of a biophysical proxy for antibody effector function.
• Antigen and Fc receptor recognition characteristics are captured.
• Enables systematic serologic studies of NHP and human antibody samples.

## 1 Introduction

Evaluation of antibody responses to vaccines has traditionally focused primarily on antibody titer, the amount of antibody present that recognizes a specific antigen or epitope. Beyond the magnitude of the antibody response, qualitative differences in the capacity of elicited antibodies to neutralize infectious particles (to prevent infection of target cells), or to agglutinate target cells (to facilitate their clearance), to fix complement, to induce opsonophagocytosis, or exhibit bactericidal activity have also been primary correlates of protection (Plotkin, 2010). As researchers focus on understanding immune pathways and developing vaccines for challenging pathogens, such as HIV, the role of other antibody functions are coming into focus as important drivers of protection or viral control (Rerks-Ngarm et al., 2009, Hessell et al., 2007, Hidajat et al., 2009, Florese et al., 2009, French et al., 2010, Ackerman et al., 2012, Xiao et al., 2010, Chung et al., 2014, Yates et al., 2014, Ackerman et al., 2013a, Ackerman et al., 2013b). These roles are dependent on the constant region (Fc) of the antibody, which can be recognized by Ig receptors on innate immune cells, and trigger several types of productive responses including antibody-dependent cellular phagocytosis (ADCP) (Ackerman et al., 2011), antibody-dependent cellular cytotoxicity (ADCC) (Gomez-Roman et al., 2006a), antibody-dependent cellular viral inhibition (ADCVI) (Forthal et al., 1999) and complement-dependent cytotoxicity (CDC) (Hezareh et al., 2001). The importance of such effector functions has long been recognized in the field of therapeutic monoclonal antibodies (Cartron et al., 2002, Liu et al., 2014, Gennari et al., 2004, Niwa et al., 2005, Pincetic et al., 2014, Taylor and Lindorfer, 2008, Clynes et al., 2000, Musolino et al., 2008, Jefferis, 2009a). Indeed, certain therapeutics, such as rituximab, are designed specifically to elicit cell-based killing as their primary mechanism of action (Glennie et al., 2007, Winiarska et al., 2011). Recent efforts have begun to focus on eliciting antibodies that protect by similar mechanisms in vaccine settings through the use of different adjuvant preparations (Moody, 2014, Moody et al., 2014, Barnett et al., 2010), delivery methods (Barouch et al., 2015), or routes of administration (Brekke et al., 2014, Singh et al., 2014, Maeto et al., 2014).

Antibody activity in vivo is a product of the combination of binding interactions of an antibody's variable (Fv) domain, responsible for recognition of the target antigen, and the constant (Fc) domain, responsible for binding to the set of Fc receptors (FcR) that link the adaptive and innate immune systems and trigger effector functions. Characteristics of each domain that contribute to therapeutic activity are easily determined for monoclonal and recombinant antibodies. However, determining these characteristics for antibodies from polyclonal clinical samples is more complicated due to the need to first affinity-purify the sample to isolate the antibodies of interest prior to interrogating their Fc characteristics. In a polyclonal setting, the binding affinity of antigen-specific antibodies to FcRs can be modulated by IgG subclass (Jefferis et al., 1994), Fc glycosylation (Jefferis et al., 1998), and avidity driven by immune complex formation or targeting of multiple epitopes. Because it is difficult for traditional biophysical methods to capture these characteristics simultaneously, numerous functional assays including those measuring neutralization, induction of ADCC, complement fixation, and phagocytosis are sometimes performed to assay clinical sera samples as a means to understand possible modes of antibody activity in vivo (Ackerman et al., 2011, Gomez-Roman et al., 2006b, Huber et al., 2006, Polonis et al., 2008). These functional readouts are the cumulative result of multiple molecular interactions; therefore, a robust, high-throughput method to co-interrogate the coordinated activity of vaccine or infection-elicited Fv/Fab activities could be instrumental to objectively profile correlates of protective immunity. Assessment of the contributions of biophysical characteristics of antibody Fv & Fc domainso both functional and clinical outcomes could provide a comprehensive landscape of the humoral immune response and better understanding of correlates of viral control or protective immunity. This sort of biophysical readout could serve as a more economical, expedient, and robust supplement or alternative to the current gold standard cell-based assays for measurements of antibody functionality.

Here we present the use of coded microsphere arrays to affinity purify antigen-specific antibodies “on bead” prior to detection of Fc characteristics via flow cytometry using fluorescent conjugates of Fc-binding proteins as detection reagents. Bead array technology allows up to 500 antigen- or even epitope-specificities to be tested in parallel, opening a path for ultra-high throughput and multi-dimensional profiling of the humoral immune response that captures information about both Fv and Fc domain characteristics simultaneously. Such arrays have been employed previously to determine the subclass of antigen-specific antibodies (Brown et al., 2012). Here this technology is adapted to gather a much wider range of functional information including direct measurements of binding to FcγRs and complement cascade-initiating proteins such as C1q (Porter and Reid, 1978) and mannose binding protein (MBL) (Arnold et al., 2006). Thus, high throughput analysis of antigen specificity can now be coupled to numerous measurements of the Fc characteristics of these antibodies, including determining their subclass as well as binding to FcγRs, complement proteins and lectins while in the antigen-bound state. As each interaction is associated with specific effector functions (for example, C1q binding with complement deposition (Moore et al., 2010)), these measurements may provide key information in understanding the functional capacity of an antibody response. Computational approaches can then be used to link these biophysical measurements to gold-standard cell-based assays of effector function (Choi et al., 2015). Efforts have also been undertaken to employ the high-throughput information gathered using this approach to profile responses to vaccination (Ilyushina et al., 2015, Wright et al., 2014) and infection (Ackerman et al., 2016), to uncover previously unknown immune correlates, and to predict protection (Vaccari et al., 2016).

## 2 Materials and methods

### 2.1 Preparation of antigen-coated array microspheres

HIV antigens were conjugated to magnetic carboxylated fluorescent beads (Luminex Corporation) as described previously (Brown et al., 2012) with minor changes. Antigens tested included proteins, peptides, and even whole inactivated virus, as described previously for poliovirus (Wright et al., 2014). Antigens highlighted in this work were mostly obtained from Immune Technologies (HIV and some SIV envelopes) or the NIH AIDS Reagent Program (HIV non-envelope proteins, some SIV envelopes). Briefly, a total of 5 million carboxylated beads (400 μL) were coupled to 25 μg of antigen using a two-step carbodiimide reaction using magnetic separation, a procedure which can be scaled down based on a ratio of 5 μg antigen per million beads. Bead storage buffer was removed via magnetic separation and the beads were washed with 100 μL of dH_2_O prior to activation for 20 min by suspension in 80 μL of Activation Buffer (100 mM monobasic sodium phosphate, pH 6.2), followed by the addition of 10 μL each of 50 mg/mL N-hydroxysulfosuccinimide (Sulfo-NHS, Pierce #24520) and 1-ethyl-3-[3-dimethlyaminopropyl]carbodiimide-HCl (EDC, Pierce #77149) dissolved in Activation Buffer. Activated beads were washed three times in 250 μL of Coupling Buffer (50 mM MES, pH 5.0), resuspended in 100 μL of Coupling Buffer, and incubated with 25 μg of antigen for 2 h on a rotational mixer. Finally, coupled beads were washed three times with 200 μL of PBS-TBN (PBS-1X, 0.1% BSA, 0.02% Tween 20, 0.05% Sodium Azide, pH 7.4) and blocked in 250 μL of PBS-TBN. After either 30 min (room temperature) or overnight (4 °C) incubation in PBS-TBN, beads were washed to remove blocking buffer and resuspended in PBS-TBN for storage. The coupled beads were counted and stored at − 80 °C for up to 6 months or at 4 °C for up to 1 month prior to use.

Minor modifications to this procedure have been made for evaluation of biotinylated proteins and peptides, and for evaluation of glycoprotein antigens with lectin detection reagents. Briefly, for biotinylated antigens, streptavidin was conjugated directly to the beads as described above, followed by a 30 min incubation with 5 μg of biotinylated antigen per million beads and three subsequent washes with 200 μL of PBS-TBN. For assessment with lectin detection reagents, antigen-conjugated beads were pretreated with PNGaseF enzyme. Conjugated beads were buffer exchanged into 20 mM Tris pH 8.2 in a 1.5 mL Eppendorf tube to a final concentration of 100 beads per type per μL. PNGase F enzyme (NEB P0704S, 500 units/μL) was then added to a final concentration of 2000 (low) or 10,000 (high) units of enzyme per million beads. The beads were incubated overnight at 37 °C with rotation, after which they were buffer exchanged into Assay Buffer prior to use.

### 2.2 Antibody samples

Human subjects were recruited from Ragon Institute cohorts and included healthy, acute, and chronically HIV infected subjects, as well as controllers, individuals able to maintain long-term suppression of virus in the absence of anti-retroviral therapy. The study was approved by the Massachusetts General Hospital Institutional Review Board, and each subject gave written informed consent. Rhesus plasma samples were obtained from an immunization study approved by the appropriate Institutional Animal Care and Use Committee. Individual plasma samples were aliquoted into 384-well master plates, and subsequently serially diluted to the desired concentration in PBS. Pooled purified immunoglobulin G isolated from HIV-infected (HIVIG, NIH AIDS Reagent Program) and healthy (IVIG, Sigma) donors were used as controls.

Subclass switched VRC-01 variants were prepared as follows. Human IgG subclass backbone plasmids were obtained from Invivogen (pFUSEss-CHIg-hG2, pFUSEss-CHIg-hG3, pFUSEss-CHIg-hG4). The entire VRC-01 variable region was cloned out of the parent pCMV VRC01 IgG1 HC plasmid (NIH AIDS Reagent Program) and inserted into each individual subclass backbone plasmid. Sequencing was performed to confirm variable region insertion. Subsequently, each VRC-01 subclass gene was cloned back into an empty pCMV plasmid to maximize dual expression of the heavy chain with the pCMV VRC01 IgG1 LC plasmid (NIH AIDS Reagent Program) (Miranda et al., 2007, Cavacini et al., 1995).

### 2.3 Preparation of IgG-Fc detection reagents

Human FcγRs (FcγRI, FcγRIIa, FcγRIIb, FcγRIIIa, FcγRIIIb) and MBL were produced via transient transfection in HEK293 cells, and purified via immobilized metal affinity chromatography (IMAC) followed by size exclusion chromatography (SEC) as described previously (Boesch et al., 2014). Size and purity of all recombinant proteins was confirmed by SDS-PAGE. Human C1q (Fischer Scientific ICN19139101) was purchased unlabeled and biotinylated according to the procedure described below. Biotinylated lectin detection reagents (SNA, ConA, GNL, MAL, LCA, RCA, PNA, AAL, VVL, and UAE1) were purchased from Vector Laboratories (B-1305, B-1005, B-1045, B-1245, B-1085, B-1075, B-1235, B-1315, B-1065, B-1395).

FcRs were chemically biotinylated using EZ-Link Sulfo-NHS-SS-Biotin (Pierce 21331) at a molar ratio of 5 mols biotin per mol of protein. Biotinylation was carried out for 2 h at RT, with a protein concentration of 0.2 mg/mL. Afterwards, excess biotin was removed via 3 rounds of dilution with PBS and concentration using 3 kD cutoff centrifugal filter units (Amicon UFC900396).

Immediately prior to use, the biotinylated FcR was mixed with a 1/4th molar ratio of Streptavadin-PE (Prozyme PJ31S), diluted to a final concentration of 1.0 μg/mL FcγR in Assay Buffer (PBS-1X + 0.1% BSA + 0.05% Tween20), and mixed for 10 min with rotation. After mixing, 1% v/v of 500 μM free biotin was added to completely block any free streptavidin binding sites. Biotinylated lectin reagents were obtained commercially from Vector Laboratories. Lectin tetramers were produced in the same manner as FcRs, except that the dilution buffer was 20 mM Tris pH 8.0 + 0.1 mM Ca ++, Mg ++, Mn ++.

### 2.4 Preparation of AVI-tagged FcγRs

Rhesus FcγRs were produced as described previously (Chan et al., 2016) and human FcγRs reformatted with a C-terminal GGG-AVI-His tag (GGGLNDIFEAQKIEWHEHHHHHH) in order to allow site-specific enzymatic biotinylation. Expression and purification were carried out as described for the non-tagged variants. Biotinylation was carried out according to the conditions of the BirA biotin-protein ligase bulk reaction kit (Avidity). Briefly, 1 mg of FcγR after SEC purification (in PBS) was diluted to ~ 40 μM. A 1/8 volume of the diluted FcγR of each reagent Biomix A and Biomix B were added to the FcγR, followed by 1 vial (10 μL at 3 μg/μL) of BirA enzyme. The reaction was allowed to proceed for 2 h at 30 °C with end-over-end mixing. After biotinylation, the reaction mixture was buffer exchanged 3 × into PBS using 3 kD cutoff centrifugal filter units (Amicon UFC900396). Biotinylated FcγRs were detected at 280 nm to determine their concentration, and stored at − 80 °C for up to 6 months prior to use.

### 2.5 CE glycan determination

The fucosylation state of antigen-purified antibodies was determined as described previously (Mahan et al., 2015). Briefly, glycan was released from purified IgG via treatment with PNGaseF and the dried glycan labeled via reductive amination. Labeled glycans were then analyzed on a 3130XL ABI DNA sequencer using POP7 polymer in a 36 cm capillary and glycan peaks labeled via comparison with a known glycan standard.

### 2.6 Fc Array protocol

Coupled microspheres were premixed in Assay Buffer, creating a working mixture of 10 microspheres per bead type, per μL. A volume of 40–45 μL of the working microsphere mixture (50 beads of each type/well) was added to 5–10 μL of serum or monoclonal antibody diluted in PBS in black, clear bottom 384-well plates (Greiner Bio One, 781,906). The optimal dilution of serum varied for different measurements and different serum samples depending on the quantity of antigen-specific antibodies present and the affinity of the detection reagent used. Multivalent interactions such as antibody-FcR or antibody-lectin typically required the greatest amount of antibody (1:100 to 1:1000 dilution from serum), while monoclonal antibody detections such as titering reagents (e.g., anti-human IgG) typically used a lower concentration (1:1000 to 1:10,000 dilution from serum). For monoclonal antibody samples (b12 and VRC01), standard curves were premade in master plates starting at 100 μg/mL in PBS and with further 1:4 dilutions. In each assay well a 10 μL volume of diluted antibody was then added to 40 μL of bead mix to result in final assay concentrations ranging from 4.8 ng/mL to 20 μg/mL. After addition of beads and antibody sample, the plate was covered and incubated for 2 h at RT on an orbital plate shaker (IKA 3208001 MTS 2/4). The plate was then washed six times with 65 μL of Assay Buffer using a plate washing system (BioTek 405). Antigen-specific antibody subclasses were detected with R-phycoerthrin (PE)-conjugated mouse anti-human IgG1–4 (9052, 9070, 9210, 9200, Southern Biotech), a murine anti-Hu pan IgG reagent (9040-09 Southern Biotech), or an R-phycoerthrin (PE)-conjugated anti-Rhesus IgG (Southern Biotech 6200-09) at 0.65 μg/mL, with 50 μL/well. Antigen-specific antibody binding to FcRs or lectins was detected using the tetrameric PE-conjugated detection reagents described above, at 1.0 μg/mL with 50 μL/well. After addition of detection reagent, the beads were resuspended via a 30 s vortex followed by 30 s sonication, repeated twice. Assay plates were then incubated for 1 h at room temperature on the shaker. The plate was subsequently washed six times with 65 μL of sheath fluid (Luminex Corp), and beads were resuspended in 40 μL of sheath fluid via two rounds of vortexing and sonication as described above immediately prior to analysis.

A Bio-plex array reader (FlexMap 3D, Bio-Plex Manager 5.0, Bio-Rad) detected the beads and PE fluorescence was measured to calculate a Median Fluorescence Intensity (MFI) for each bead type. Data from 30 beads per type per well was collected and analyzed. HIV-IG (3957, AIDS Reagent Program) was used as a positive control and a means to track plate-to-plate variation, and Human IgG (I2511, Sigma) served as an HIV-1 negative control.

### 2.7 Data analysis

Raw data were analyzed in the Bio-Plex Manager 5.0 software for assignments of standard, controls, samples, etc. Data were exported as Excel files and input into GraphPad Prism for analysis. For determining binding coefficients, the MFI and antibody concentration were log transformed and fit with a non-linear regression using the Variable slope (four parameter) function. EC_50_ values were extracted from the curve fit and converted to antibody concentrations on a nanomolar scale. For comparison of array signals with previously determined ELISA and Surface Plasmon Resonance (SPR) affinity data, the raw array MFI was log-transformed and plotted against a log-transform of the published binding fold-change determined either by ELISA or SPR (Moldt et al., 2011). The log-transformed fold changes of ELISA and SPR binding were also directly compared to each other as a reference. For specificity testing, MFI signals from unvaccinated and vaccinated groups were compared in Prism by *t*-test with Welch's correction for unequal variances.

Heatmaps and PCA (principal component analysis) plots were generated in R version 3.02. In order to facilitate comparisons among the different features (antigen:detection reagent pairs), data was normalized by centering to a mean of 0 and scaling to a standard deviation of 1 for each feature. The function heatmap.2 in the R gplots library was then used to hierarchically cluster and visualize the data, grouping and reordering features and samples by overall similarity. The PCA analysis function prcomp was used for generating lead PCs.

## 3 Results

### 3.1 Method overview

**Fig. 1:**
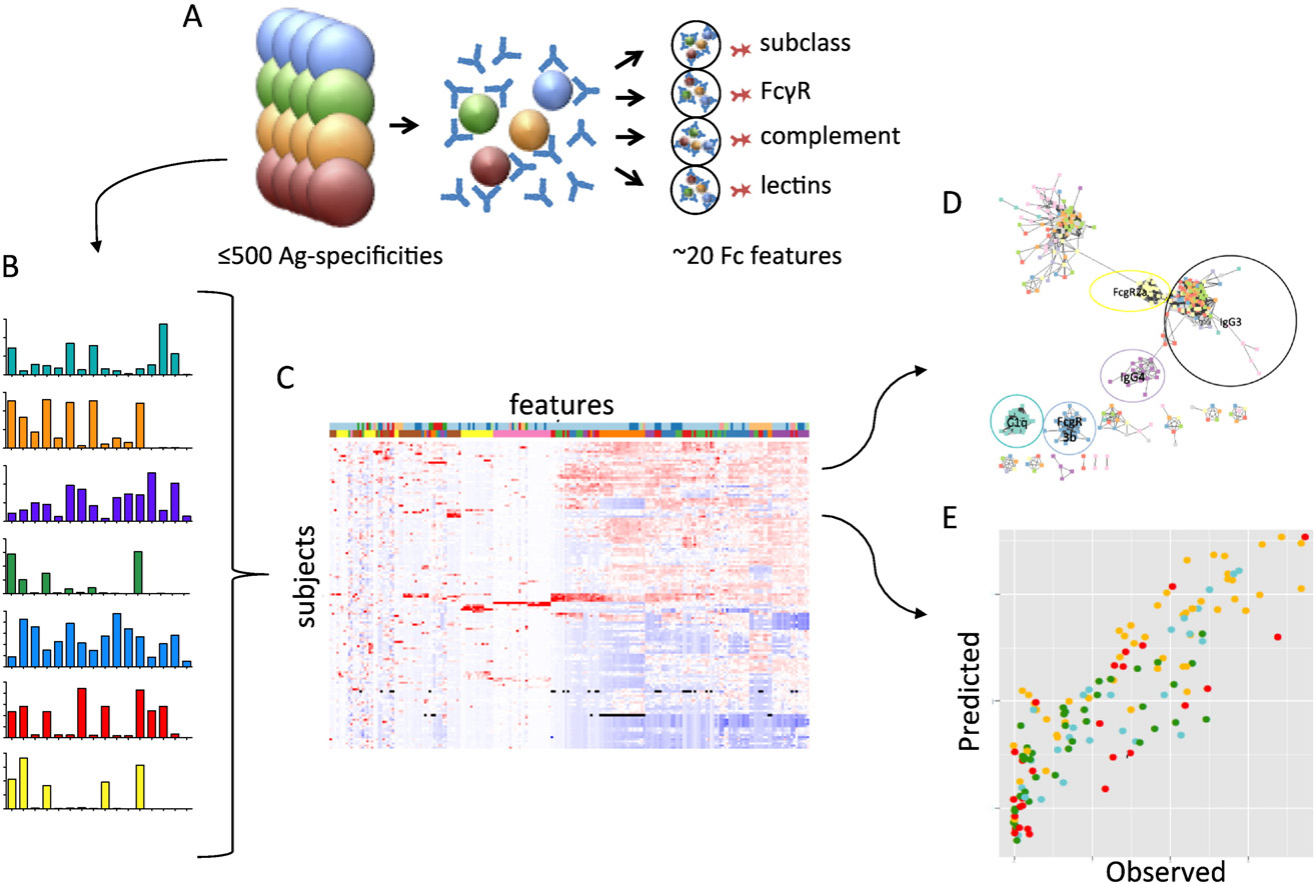
**Fc Array Assay Schematic**. **A**. HIV (or other) antigens are conjugated to fluorescently-coded magnetic beads, incubated with serum or other polyclonal Ig samples, capturing antibodies specific to each antigen of interest prior to characterization with a panel of Fc-specific detection reagents such as FcγRs. **B**. Median Fluorescence Intensity (MFI) data is collected on the array reader for each specificity and subject. **C**. Output data from multiple detection reagents are combined to give a profile of the humoral response across features and subjects. The combined data can be used as the input features for computational analysis including grouping of features (**D**) and predictions of functional assays (**E**).

The goal of this project was to develop a method to more comprehensively map the breadth and functional characteristics of the humoral immune response to HIV infection and/or vaccination. To this end, multiple HIV antigens of interest were used to obtain a profile of the breadth of the antibody response, including surface antigens (gp120, gp41, gp140) from many different clades, as well as structural antigens (p24) and other internal viral proteins (Rev, Nef, Pol, Integrase, Vif). To demonstrate the adaptability of the Fc Array to other settings, preliminary experiments were also conducted with antigens from influenza (hemagglutinin and neuraminidase) (Ilyushina et al., 2015), polio (whole virus) (Wright et al., 2014), TB, and Hepatitis C, among other pathogens. As diagrammed in Fig. 1A, individual antigens were conjugated to fluorescently coded magnetic beads with known concentrations of three dyes, that permit up to 500 different antigen specificities to be differentiated simultaneously. These antigen-coupled beads were then incubated with IgG from clinical samples, allowing affinity purification of antigen-specific antibodies “on bead” for each antigen. Beads were subsequently washed, and incubated with R-phycoerythrin conjugated detection reagents which interrogate either total IgG, individual IgG subclasses, or Fc characteristics such as N-linked glycosylation (lectins) and functional capability (binding to FcγRs and complement proteins). The beads were then analyzed via multiplex flow cytometry in which each antigen bead was uniquely identified by fluorescence in red and IR channels, and the amount of Fc-binding reagent determined by the fluorescence intensity observed in the orange channel (Fig. 1B). Output data can be combined for multiple detection reagents to create a full data set for a group of samples (Fig. 1C), and then used as input for computational analysis. Computational analysis used include both unsupervised methods (Fig. 1D), such as hierarchical clustering and Principal Component Analysis (PCA), which aim to reveal non-obvious patterns and relationships in high-dimension data that may be missed in lower-dimensional analysis, as well as supervised methods (Fig. 1E) such as Regularized Random Forest (RRF) and Least Angle Regression (LARS), which attempt to predictively model outcomes or parameters of interest based on previous observations (Choi et al., 2015).

### 3.2 FcR comparison to current methods

**Fig. 2:**
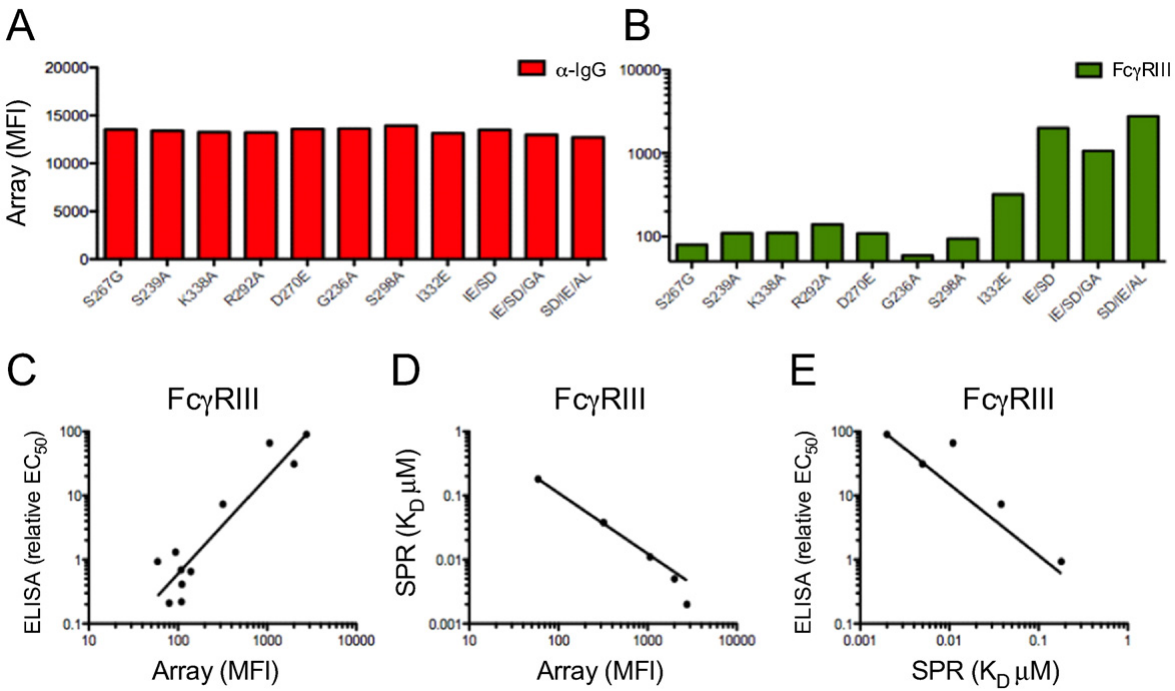
**Comparison with gold standard FcR affinity characterization by SPR**. Comparison of previously determined ELISA/SPR binding affinities (Moldt et al., 2011) of a panel of mAb Fc mutants against Fc Array MFI. **A)** Anti-huIgG-PE detection of Fc variants bound to gp120-conjugated beads. **B)** FcγRIII detection of Fc variants bound to gp120 beads. **C-E)** Correlations between ELISA and Fc Array **(C)**, SPR and Fc Array **(D)**, and ELISA and SPR **(E)** for the panel of Fc mutants.

In order to confirm the validity of FcγR detection reagents, a series of b12 (anti-gp120) monoclonal antibodies with Fc mutations designed to have different affinities to FcγRs (Moldt et al., 2011) was evaluated in the Fc Array. These monoclonal antibody variants were incubated with gp120-coated microspheres at equal concentrations and measured with tetrameric FcγRIII detection reagent, as well as an anti-huIgG-PE detection reagent (Fig. 2A–E). Binding of the monoclonal antibodies was very consistent across all variants tested, as measured by the tittering antibody, with a total %CV of 2.5% of raw MFI (median fluorescent intensity) signal between variants tested (Fig. 2A). In contrast, the FcγRIII signal varied considerably (2.5 orders of magnitude) between variants (Fig. 2B) and agreement between the observed binding levels (MFI) measured by the Fc Array and previously determined binding affinities of these monoclonal variants by ELISA and SPR (Moldt et al., 2011) was excellent (Fig. 2C–D). Interestingly, the agreement between array and published SPR binding affinities was superior to that observed between the ELISA and SPR data published previously (Fig. 2E).

### 3.3 Fc-receptor challenges and solutions

Several challenges were experienced in the expansion of the array to detect binding with FcRs such as FcγRs and C1q as opposed to the commercially available antibody-based detection reagents used in prior work (Brown et al., 2012). First, most of the FcγRs tested (FcγRII and FcγRIII alleles) have very low monovalent affinities (in the micro-molar *K*_D_ range) (Lazar et al., 2006, Radaev and Sun, 2002), and are characterized by off rates that are considerably faster than the timescale of the assay itself. We therefore constructed PE-conjugated streptavidin-FcR tetramers to act as polymeric detection reagents. Importantly, receptor multimerization also recapitulates natural FcR biology, which is highly dependent on avid interactions; indeed multimerized (in this case dimeric) FcγRs have been shown to be useful for detection of antigen-bound antibody (Wines et al., 2016). This procedure involves two steps, biotinylation of the FcR of interest followed by co-incubation with streptavidin-PE detection reagent in a 1:4 M ratio. Bio-layer interferometry experiments demonstrated that these FcR tetramers showed much slower off rates than monomers (data not shown). In order to test the effect of reagents' off-rate on assay results, HIVIG standard curves were run in different wells of a 384w plate with a 3 h temporal separation (the maximum possible for a single plate) between reads. The two resulting time-separated titration curves were comparable (**Supplementary** Fig. 1), demonstrating that tetramerized FcγR detection reagents have dissociation rates that are sufficiently slow to enable reliable measurement across an entire 384-well plate.

Initially, primary amine-based biotinylation (EZ-Link™ Sulfo-NHS-Biotin, Thermo) was used, but later AVI-tagged constructs of the FcγRs were constructed to take advantage of the high specificity biotin ligase, birA (Chapman-Smith and Cronan, 1999). Over-biotinylation using the amine-based biotinylation tended to reduce binding, presumably through biotinylation of lysine residues known to be near the Fc binding site of some FcRs (Mizushima et al., 2011). In contrast, using an enzymatic system ensured that a single biotin was attached at a defined site, which tended to give more efficient formation of active tetramers and yielded better signal and reproducibility.

### 3.4 FcR detection reagent dynamic range and specificity

For further confirmation of their suitability, these tetramerized FcR reagents were probed in the Fc Array Assay for dynamic range and sensitivity across a HIVIG standard curve to generate an approximate dynamic range. While antibody detection reagents used in the previous work (Brown et al., 2012) had dynamic ranges of > 4 orders of magnitude, as illustrated by the anti-human IgG trace, the tetramerized FcR detection reagents tended to have a somewhat narrower dynamic range of 2–4 orders of magnitude (**Supplementary** Fig. 2). Because only the antibody specific to the antigen on the bead is being assayed, the amount of antibody needed varies with titer against the specific antigen or antigens of interest, and thus the dynamic ranges shown here are representative only.

**Fig. 3:**
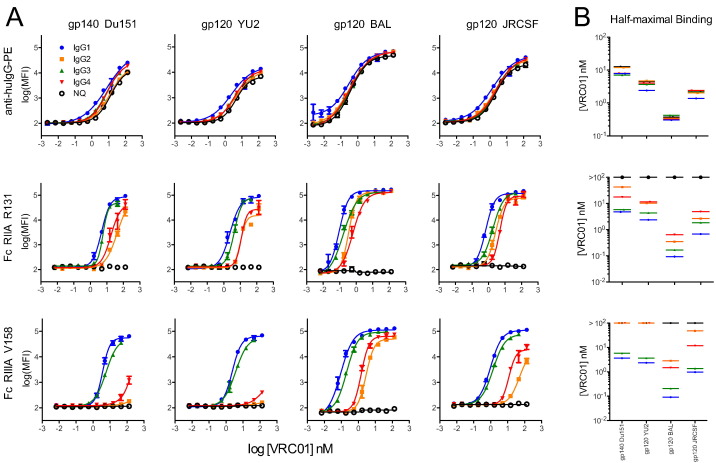
**Differentiation of IgG subclasses and an aglycosylated mutant via FcγR detection**. **A**) Titration curves of subclass switched and aglycosylated (N297Q, or NQ) VRC01 Abs against HIV envelope beads detected with anti-huIgG-PE to quantify the level of mAb binding to each envelope variant, or detected with FcγRIIA R131 or FcγRIIIA V158 to quantify the ability of bound mAb to interact with these innate immune receptors. **B)** Calculated half-maximal binding concentration of each VRC01 variant to each antigen-conjugated bead, for each Fc detection reagent shown in (**A**). Curves that could not be reliably fit were plotted with values at the top of each scale.

As a further test of the utility of the FcγR detection reagents, we evaluated a set of subclass switched VRC01 anti-gp120 monoclonal antibodies including IgG1/2/3/4 and IgG1-N297Q variants. The different IgG subclasses are known to have varying affinities for the FcγRs (Bruhns et al., 2009, Nimmerjahn and Ravetch, 2005), and the N297Q mutant removes the Fc N-glycosylation site, which greatly reduces binding to low affinity FcγRs (Shields et al., 2001, Tao and Morrison, 1989) and is known to decrease effector functions such as ADCC in vitro and in vivo (Stavenhagen et al., 2007). A dilution series of each variant was incubated with a set of HIV Env-conjugated beads (gp140 Du151, gp120 YU2, gp120 BAL, and gp120 JRCSF) and detected separately with an anti-human IgG-PE and two tetrameric FcγR-streptavidin-PE detection reagents (FcγRIIA-R131 and FcγRIIIA-V158) (Fig. 3).

For all antigens tested, the anti-human IgG-PE signal (MFI) showed very good agreement across all VRC01 subclass variants (Fig. 3A). In contrast, subclass- and glycan-dependent differences were observed among the FcγR detection reagents (Fig. 3A), as expected. Half-maximal binding concentrations were calculated for each curve (Fig. 3B). In line with expected results, the IgG1 and IgG3 variants demonstrated the tightest binding, IgG2 and IgG4 weaker binding, and the N297Q mutant no detectable binding to the FcγRs used. Interestingly, the pattern of binding for the subclass variants to FcγR tetramers appeared to shift across the different HIV envelopes tested, indicating that differences in the antigen can result in differences in the spatial presentation of the antibody, and may play a role in the ability of these antibodies to engage FcγRs. In particular, the binding of FcγRIIIA V158 to VRC01 IgG2 and IgG4 complexed with gp140 Du151 and gp120 YU2 beads appeared to be more severely ablated as compared to the ability of the same antibodies to engage the FcγR when bound to the other HIV Envs (Fig. 3A).

### 3.5 Lectin challenges and solutions

**Fig. 4:**
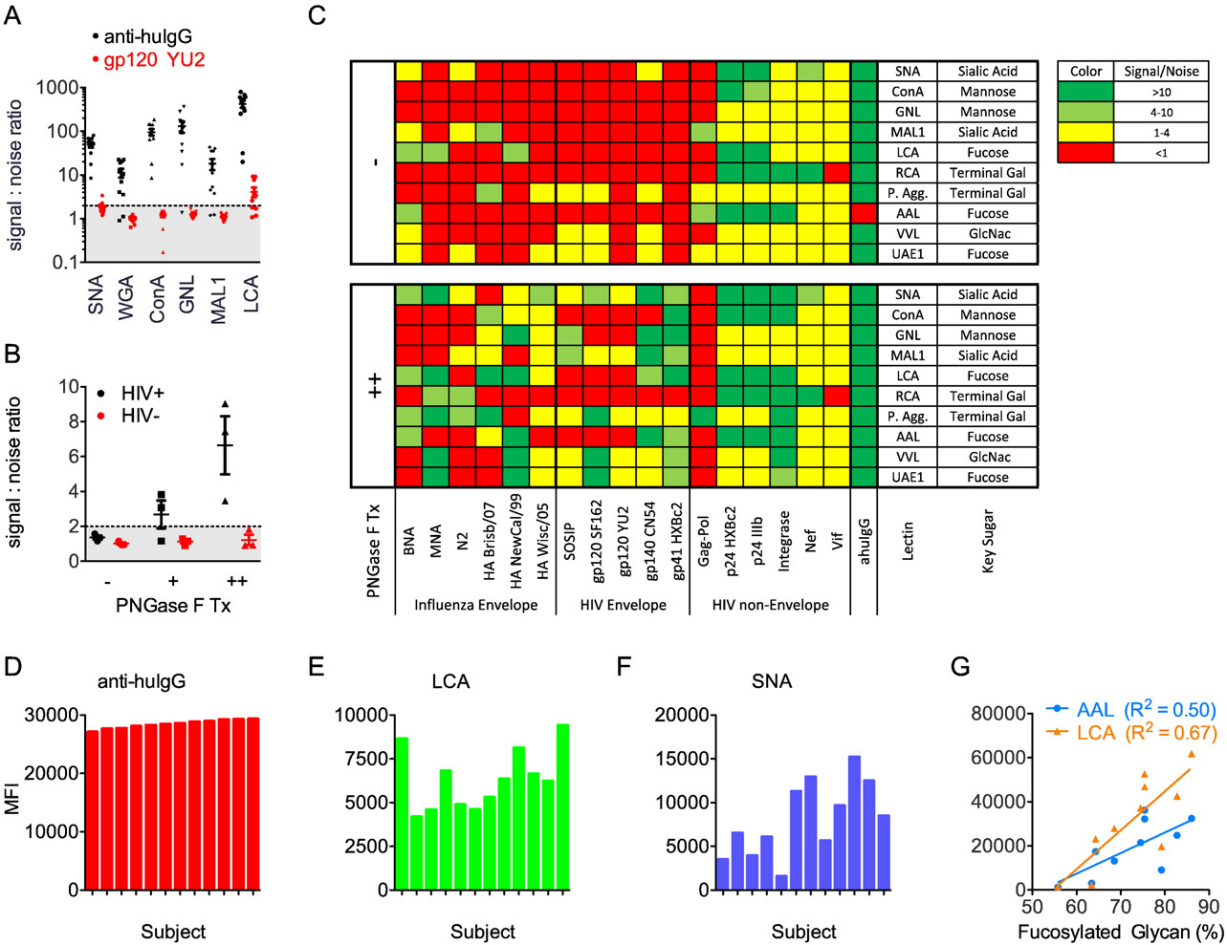
**IgG glycosylation state differences captured by lectin detection reagents**. **A**) Signal to noise ratios of lectin detection reagents for antibody samples from HIV + subjects (N = 11) against a technical negative (blank) on anti-huIgG and gp120 YU2 conjugated beads. Shaded region denotes values with a signal:noise ratio of < 2. **B**) Signal to noise ratios of AAL lectin detection for HIV positive and negative subjects (N = 3) on gp120 CN54 beads with and without prior PNGase treatment. Shaded region denotes values with a signal:noise ratio of < 2. **C**) Overview of PNGase treatment of conjugated beads. Signal of HIVIG compared to a technical negative (PBS) sample against beads conjugated with the named antigens before and after PNGase treatment and detected with tetramerized lectin detection reagents. **D–F**) Observed signal intensities (MFI) for a set of twelve subject samples with similar responses against gp120 CN54 when detected with total anti-huIgG-PE (**D**) but differing response magnitude when assayed with the fucose-specific lectin detection reagent LCA (**E**), and the sialic acid-sensitive lectin SNA (**F**). **G**) Correlation between signals (MFI) from fucose-specific lectin detection reagents (AAL and LCA) on anti-huIgG beads and the prevalence of fucosylated glycoforms from total serum IgG for a set of 10 subject samples.

A more fundamental challenge arose when attempting to use lectins as detection reagents. Many of the antigens of interest, in particular the HIV envelope components, are highly glycosylated; therefore, significant antigen-lectin background signal was observed in the absence of antibody sample, resulting in a low signal to noise ratio for antibody–lectin binding (Fig. 4A). Two solutions were used to partially circumvent this challenge. One strategy was to enzymatically deglycosylate the antigens on the beads immediately prior to use using varying concentrations of PNGase-F. The higher of two PNGase concentrations tested (10,000 units of enzyme per million beads) gave good reduction of background and improvement in signal to noise ratio for some antigens tested (Fig. 4B). However, this method was incapable of completely removing the background signal, especially for some HIV Env variants. A second complementary approach was to downselect the antigen bead sets chosen to have the lowest background lectin binding after PNGase treatment. After optimizing the on-bead deglycosylation protocol, lectin-antigen interactions were screened and a subset of those with the highest signal to noise ratio were selected to use in further lectin studies. Signal-to-noise ratio was calculated by comparing HIVIG signal with that of a technical negative (PBS). A summary of these results is shown in Fig. 4C.

One of the most important glycoforms to measure is antibody fucosylation, which has been shown to be of critical importance for binding to FcγRIIIa and thus ADCC activity of antibodies (Tao and Morrison, 1989, Wright and Morrison, 1997, Jefferis, 2009b, Shinkawa et al., 2003). To test the efficacy of lectin detection reagents in detecting fucosylation levels, a set of gp120-titer matched patients was assayed by anti-huIgG-PE, the fucose-preferring lectin LCA (Kornfeld et al., 1981), and the sialic acid sensitive lectin SNA on the gp120 CN54 bead after deglycosylation. The output MFIs showed wide variation in lectin signal despite essentially identical signal from the anti-huIgG-PE reagent (Fig. 4D–F). In another experiment total polyclonal IgG was detected with fucose-preferring lectins LCA and AAL (Yazawa et al., 1984) for a set of samples with known fucose content determined by capillary electrophoresis (Mahan et al., 2015). For both fucose-preferring lectins, a strong positive correlation (R^2^ = 0.50 for AAL and 0.67 for LCA) of overall % fucosylation with fucose lectin readouts (Fig. 4G) was observed.

### 3.6 Extension to non-human primate (rhesus) reagents

Translating the Fc Array assay for use with NHP subjects, particularly rhesus macaques, was of critical importance because of the role these animals play in HIV vaccine preclinical studies (Hessell and Haigwood, 2015). Despite the importance of the model, the two systems are not perfectly analogous. For example, macaques are vulnerable to a very closely related virus (simian immunodeficiency virus or SIV (Reimann et al., 1996)) but not to HIV itself. In addition, while there is certainly functional and structural homology between species (and receptor orthologs are named consistently across species) the Fc detection reagents developed for the human Fc Array assay were not ideally suited for NHP work owing to small but significant differences in the structure of macaque antibody subclasses and the Fc receptors that recognize them (Chan et al., 2016, Warncke et al., 2012, Hogarth et al., 2014, Boesch et al., 2017, Boesch et al., 2016). These distinctions necessitated the development and testing of novel antigen-bead pairs using SIV-derived antigens and rhesus FcγR variants.

**Fig. 5:**
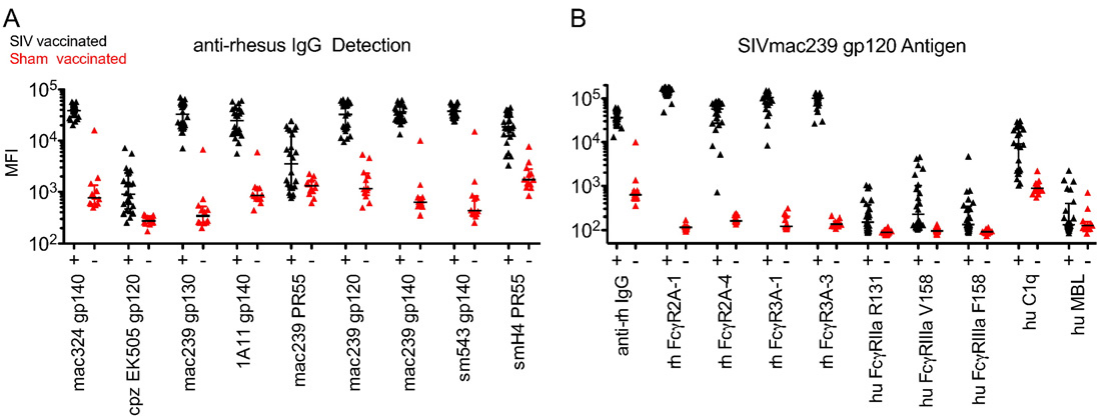
**Specificity of rhesus antigens (A) and detection reagents (B) for NHP samples**. **A**) Reponses of SIV vaccinated (+, black) and sham (−, red) macaques against a panel of SIV antigens using the anti-rhesus IgG-PE detection reagent. Bar and whiskers denote the median and interquartile ranges for each group. **B**) Responses of the same SIV vaccinated (+) and sham (−) macaques against a single antigen (SIVmac239 gp120) detected with nine different human (hu) and rhesus (rh) derived Fc-binding detection reagents in addition to the anti-rhesus IgG-PE antibody.

Rhesus FcγR variants were constructed with AVI-tags and produced in-house in a manner analogous to the human FcγRs described previously. To test the specificity of these reagents, a set of 36 samples was selected from an SIV vaccine study, in which 24 animals were vaccinated according to two different regimens, and twelve animals were sham controls. These samples were uniformly diluted to a single concentration (1:1000 total dilution from serum) and evaluated against nine commercially available SIV antigens and with 10 different Fc detection reagents. Detection reagents included an anti-rhesus titering antibody (anti-rhesus IgG-PE), rhesus (rh) and human (hu) FcγR variants, as well as human mannose binding lectin (MBL) and C1q, FcRs for which the corresponding rhesus variants were not readily available. Responses toward most antigens showed highly significant differences in sample means (p-values < 0.0001) between vaccinated and unvaccinated macaques using anti-rhesus IgG-PE (Fig. 5A), with the exception of an envelope from a chimpanzee-derived strain (cpzEK505 gp120) and the two PR55 variants tested. Rhesus FcR variants showed good differentiation of vaccinated and control samples across most antigens tested (Fig. 5B). While the sensitivity observed with human FcγRs was lower, species mis-matched data may be useful in translational studies, or in cases in which the goal is to predict the output of a functional assay carried out with human effector cells.

### 3.7 Characterization of an HIV-infected cohort

**Fig. 6:**
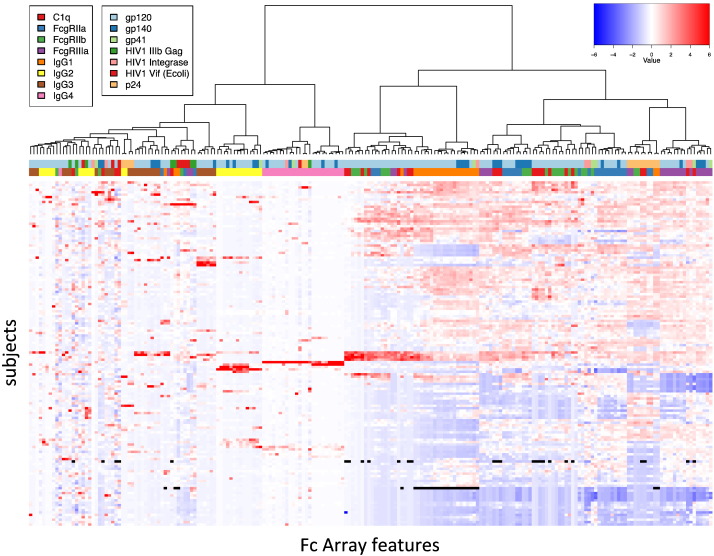
**Similarities and differences among Fv-specificity and Fc characteristics across a cohort of HIV + human donors**. A heatmap and dendogram of data centered to the mean and scaled by standard deviation for each feature showing the relationship of each of the Fc Array features (antigen-detection reagent pairs as indicated in color bars) across a cohort of HIV-infected subjects. Hierarchical clustering has grouped features and subjects by similarity. Missing data points are indicated in black.

The true utility of this method is its high throughput and ability to profile both the variable and constant domains of the immune response simultaneously. An example of the data generated from one such analysis is shown in Fig. 6. For this experiment, purified IgG samples from a set of 152 HIV-infected and uninfected patients were assayed for binding to 32 antigens (28 HIV and 4 control) and detected with 9 Fc-binding detection reagents, including titering Ab (anti-IgG), subclassing antibodies (anti-IgG1–4) human FcγRs and human C1q. These subjects included elite controllers (< 50 copies RNA/mL), viremic controllers (50–200 copies RNA/mL), HIV-positive patients on anti-retroviral therapy (< 50 copies RNA/mL) and untreated HIV-positive patients (> 50 copies RNA/mL). A heatmap of antibody features (antigen-detection reagent pairs) versus subjects was generated showing subjects clustered on the y-axis, and antigen-detection reagent features clustered on the x-axis. Clusters of subjects can be seen with differing antibody signatures, such as small groups with high IgG4 or FcγRIIb signal across multiple antigens in the center of the plot, and a large cluster with high FcγRIIa, FcγRIIIa or FcγRIIa and FcγRIIIa signal in the bottom right region of the plot. It can be readily observed that response variables cluster both on antigen and detection features, indicating that both interactions differ between subjects and are each being assessed effectively. Signal for many detection reagents was correlated with titer, since if there is not antibody present against a particular antigen there can be no detection, but in positive samples the signal also modulated by Fc characteristics such as subclass and glycan.

### 3.8 Contributions of Fc and Fv measurements

**Fig. 7:**
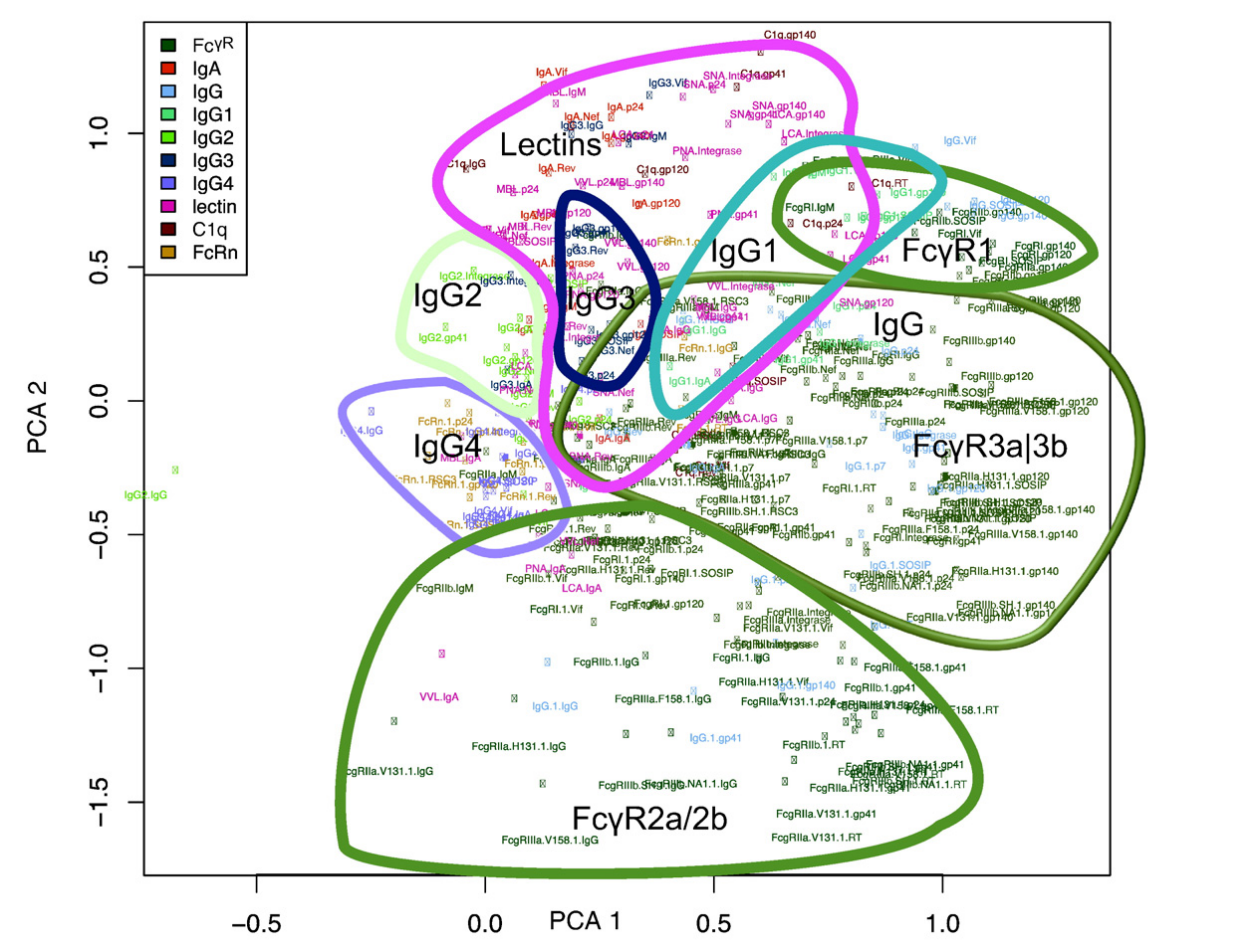
**Principal Component Analysis identifies shared Fc characteristics as differentiating among HIV infected subjects**. A principal component (PC) biplot indicating the magnitude and direction that each Fc Array feature contributed to the two lead PCs. Features are color coded by detection reagents and indicated by dots labeled with “detection reagent.antigen” combinations. Groupings were manually drawn to showcase Fc-dependent feature sets.

Principal Component Analysis (PCA) was performed on this dataset to identify key aspects of variation in Fc Array features (Fig. 7). Such unsupervised dimensionality reduction methods are particularly useful for Fc Array data sets where numerous Fc measurements of the same bound antibodies, and numerous closely-related HIV envelope antigens are investigated. Interestingly, the two lead PCs identified that much of the variation observed among subjects is linked to groups of features defined by Fc, as opposed to Fv, characteristics. This linkage is apparent based on the grouping of features by detection reagents as well as by antigen specificity. Both Fc and Fv characteristics have the potential to drive variation in patient responses and have predictive power for functional outcomes and thus measuring them simultaneously allows uncovering characteristics of the humoral response that would not be apparent from observing at Fv binding alone.

## 4 Discussion

The importance of effector function in control of and protection from disease has been repeatedly demonstrated across many different systems (DiLillo et al., 2014, Bournazos et al., 2014a, Benhnia et al., 2013, Bournazos et al., 2014b, Kapur et al., 2014). Prior attempts to measure these functions have generally focused on resource and time intensive cell-based assays measuring features such as ADCC, ADCP, CDC and ADCDC, which can yield relatively variable results, and do not allow inspection of the linkage between specific types of antibodies and specific antibody activities. These assays also have an inherent limitation in the breadth of antibodies which can be tested, as each generally relies on the expression or coating of a single surface antigen to the target cell or bead. For diseases like HIV or influenza for which heterologous protection is a key factor (Barouch, 2008, Pica and Palese, 2013), owing to the immense diversity of the target virus (Gaschen et al., 2002, Walker and Korber, 2001, Wright and Webster, 2001), this restricted focus can be a serious limitation to optimally evaluating efficacy. Furthermore, different versions of each of these assays are routinely performed in different laboratories sometimes leading to differences in outcomes or conclusions.

The Fc Array attempts to complement these assays and circumvent some of their limitations by utilizing a biophysical platform to simultaneously evaluate the breadth and effector potential of the humoral immune response, at high throughput and with minimal sample requirement. With this system, the ability of antibodies present in clinical sera to bind many different antigens can be probed while Fc characteristics likely to affect effector functions, such as subclass and ability to bind FcRs are assessed. In effect the goal is to mimic the presentation of antigen and thus bound antibody on the surface of a particle (in this case a bead instead of a virus or virally-infected cell) that effector cells would encounter in the body, but in a more controlled fashion than in cell-based assays. This system for evaluating FcR binding ability is similar in principle to recent work using dimeric FcγRs as probes for Fc function in influenza-specific antibodies (Wines et al., 2016), in which it was observed that effector cell activation was correlated with binding of dimeric FcγR but not with antibody titer. Here, however, bead multiplexing allows for sensing of many antigen specificities simultaneously. A similar assay to assess C1q-ligating activity of anti-donor HLA-specific antibodies has been investigated in the setting of organ transplantation (Chin et al., 2011) and demonstrated better performance than either functional or antibody titering assays in identifying subjects with or at risk for antibody-mediated transplant rejection (Chen et al., 2011, Fontaine et al., 2011, Sutherland et al., 2012, Yabu et al., 2011).

However, given that effector cells often express combinations of FcRs, and may respond differently to immune complexes of different sizes, future assays could attempt to capture these effects by varying bead size, or antigen density on the surface of the bead, or by using FcR tetramers composed of combinations of FcRs that reflect those present on different effector cell types. Modifications such as these, aimed at better capturing additional aspects of effector and target cells/particles, could provide enhanced resolution of antibody bioactivities on the surface of effector cells when they interact with immune complexes with different characteristics.

These previous studies and our current results have demonstrated both the potential clinical value and the comparability of this method with current gold standard biophysical measurements such as ELISA, SPR, and cell-based assays but with much higher power due to the multiplexed nature of the assay. For example, the assay readily identified the expected pattern of functional assays for monoclonal antibodies with variations known to affect FcR binding, such as subclass and the presence or absence of glycan. Use of FcγR reagents to probe multiple antigens simultaneously has also offered tantalizing glimpses of the effects of antigenic differences on antibody presentation and thus the ability to bind FcRs and elicit effector function, even when a titering detection reagent showed that a similar amount of antibody was bound. The effect of presentation on FcγR binding appeared particularly pronounced with the lower affinity IgG2 and IgG4 subclasses, as was noted in a prior study (Wines et al., 2016).

A key advantage of the Fc Array is that as a biophysical method these data can be generated in a highly robust and reproducible fashion, with relatively minimal investment in instrumentation and operator training, and very minimal sample requirement. Intra-assay variation has been shown to be minimal with average % coefficients of variations below 10% in a wide variety of settings. Reproducibility has been shown to be excellent across numerous assay features including operators, instruments, bead conjugation batches and even different test sites. The assay is also highly amenable to robotic sample handling to further increase throughput if needed, with sets of many hundreds of subject samples able to be analyzed in a single day. Sample requirements generally do not exceed 10 μL of patient serum to enable up to 500 Fv specificities and > 20 Fc characteristics, which would result in 10,000 data points per sample.

The use of this assay is not limited to the HIV/SIV disease model nor the FcRs so far tested. While this approach is particularly useful for HIV because of the immense variability of the HIV envelope antigen, there are numerous other diseases, including influenza, hepatitis and autoimmune diseases such as rheumatoid arthritis, where the ability to profile the effector response against a wide set of antigens simultaneously would be useful. Beyond the canonically important FcγRs and complement proteins described here, a host of less-well studied FcRs may have important functions including, but not limited to, dendritic cell-specific intercellular adhesion molecule-3-grabbing non-integrin (DC-SIGN) (Anthony et al., 2008), FcR-Like receptors (FcRLs) (Wilson et al., 2012, Wilson et al., 2010), TRIM21 (Keeble et al., 2008), macrophage mannose receptor (MMR) (Dong et al., 1999), and Dectin-1 (Karsten et al., 2012), which could easily be included in this assay to generate novel insights. In addition, the ease of conversion of such an assay between human and NHP reagents naturally points toward the inclusion of FcRs from other common animal models with known differences in FcγR structure (Guilliams et al., 2014, Lux and Nimmerjahn, 2013). Use of these animal FcγRs may even be more predictive than cell-based measures of effector function in animal models, which often utilize human-derived effector cells and thus mismatched FcγRs to those of the animal itself.

The multi-dimensional data generated by this assay can most powerfully be used as a large set of input features for modeling through machine learning methods. Unsupervised methods such as clustering and PCA can be utilized to capture key global similarities and differences between subjects or between antibody response features; supervised methods such as classification or regression can be used to identify the response features that may effectively differentiate different subject groups or among responses with variable effector function or protective efficacy (Barouch et al., 2015, Choi et al., 2015, Ackerman et al., 2016). Thus, the potential uses for this system are numerous and varied. Analysis of HIV clinical cohorts has been carried out previously using the subclassing array in an effort to identify key differences in clinical status (Elite controller, viremic controller, chronic treated, chronic untreated) (Ackerman et al., 2016), and these efforts have been extended with the full Fc Array. Analyses of these data have also been used for predictions of functional outcomes such as ADCC or ADCP with high degrees of predictive accuracy. For these predictions the key features are often antigen-Fc reagent pairs that have functional relevance rather than crude titer measurements, for example several FcγRIII-Env features served as the top predictive features for models of ADCC activity (Brown et al., 2013). In addition, analyses of HIV, SIV, and SHIV vaccine trials have begun to differentiate responses between variably protected animals, or variable responses between vaccine arms, such as those caused by differing adjuvants or routes of administration (Barouch et al., 2015, Vaccari et al., 2016). With correlates of protection identified, future vaccines could potentially be screened with shortened clinical trials. Thus the early-stage pipeline for vaccines could potentially be narrowed to focus on those with the highest chance of success based on results of NHP studies. The ultimate goal of such an analysis is not only to determine correlates of protective efficacy and predict protection in the context of HIV/SIV responses, but to more broadly characterize and thus define conserved features of functionally potent immune responses. Such features have the potential to provide mechanistic insights into composite activity of polyclonal antibody pools, and antibody feature-function relationships that are relevant for monoclonal antibody therapy.

## Appendix A Supplementary data

Supplementary figures Image 1
