# Supplementary material for: Multiplexed Fc array for evaluation of antigen-specific antibody effector profiles
Source: J Immunol Methods. 2017 Apr;443:33–44. doi: 10.1016/j.jim.2017.01.010 (PMC5333794; doi:10.1016/j.jim.2017.01.010)
Supplement: Supplementary file 1 — Supplementary figures [file mmc1.pdf]

Supplementary Figures

Brown et al., "Multiplexed Fc Array for Evaluation of Antigen-Specific Antibody Effector Profiles"

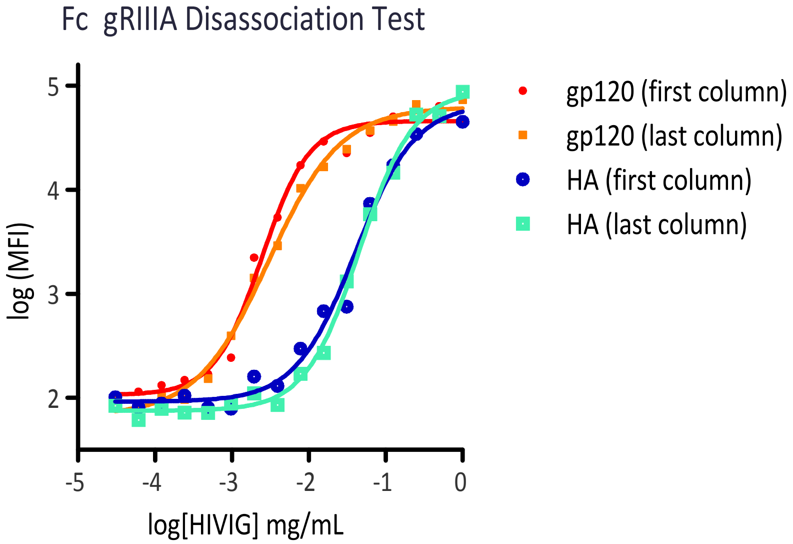

Supplementary Figure S1: Dissociation of tetrameric FcγR Reagents: FcγRIIIA detection of a HIVIG standard curve carried out in the first and last columns of a 384 well plate with a time difference of 3hrs between the instrument reading the first and last columns. Data shown from an HIV gp120 and an influenza HA antigen.

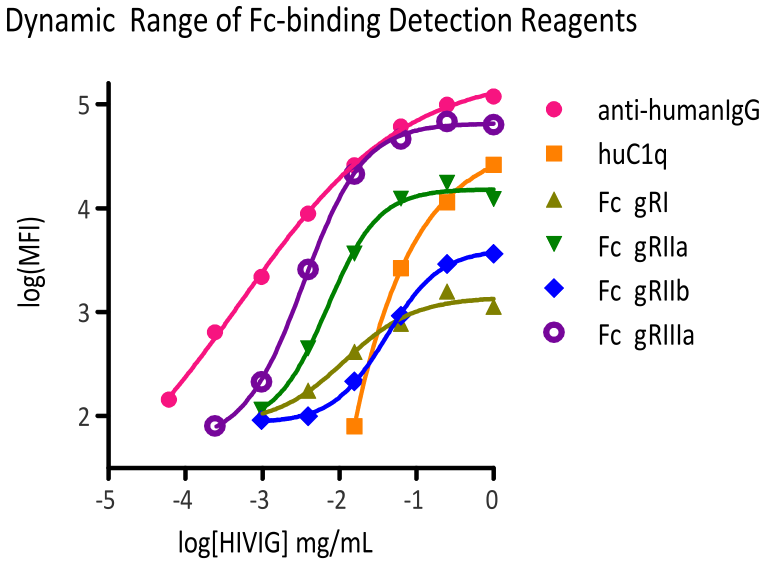

Supplementary Figure S2: Dynamic Range of Fc-binding detection reagents: Standard curve of HIVIG bound on gp120 YU2 beads and detected with various Fc-binding detection reagents. Curves truncated at concentrations where the MFI goes within three standard deviations of the background (blank) MFI for that detection reagent.
